# Supplementary material for: Evolution of the locomotor skeleton in Anolis lizards reflects the interplay between ecological opportunity and phylogenetic inertia
Source: Nat Commun. 2021 Mar 9;12:1525. doi: 10.1038/s41467-021-21757-5 (PMC7943571; doi:10.1038/s41467-021-21757-5)
Supplement: Supplementary file 1 — Supplementary Information [file 41467_2021_21757_MOESM1_ESM.pdf]

Supplementary information

Evolution of the locomotor skeleton in *Anolis* lizards reflects the interplay between ecological opportunity and phylogenetic inertia

Nathalie Feiner, Illiam S.C. Jackson, Edward L. Stanley and Tobias Uller

Supplementary Notes

Supplementary Note 1

Supplementary Figures

Supplementary Fig. 1 Quantification of variation in the locomotor skeleton.
Supplementary Fig. 2 Distribution of principal component scores by biogeographic group and ecomorph.
Supplementary Fig. 3 Negligible effect of subsampling on estimates of modularity, but substantial effect on estimates of integration.
Supplementary Fig. 4 Patterns of phenotypic integration between the four main blocks of the locomotor skeleton across the major biogeographic groups of *Anolis* lizards.
Supplementary Fig. 5 Variation in Evolutionary rates.
Supplementary Fig. 6 Ancestral state reconstruction of biogeographic groups.

Supplementary Tables

Supplementary Table 1 Anatomical position of landmarks used to quantify the shape of pectoral and pelvic girdles.
Supplementary Table 2 Loadings of the principal components.
Supplementary Table 3 Morphological disparity of girdles (108 girdle shape traits) and comparisons among biogeographic groups.
Supplementary Table 4 Morphological disparity of limbs (15 limb length traits) and comparisons among biogeographic groups.
Supplementary Table 5 Variation explained by ecomorph.
Supplementary Table 6 Statistical support for alternative modularity hypotheses in the Primary Mainland group using covariance ratio effect sizes (CR z-scores).
Supplementary Table 7 Statistical support for alternative modularity hypotheses in the Secondary Mainland group using covariance ratio effect sizes (CR z-scores).
Supplementary Table 8 Statistical support for alternative modularity hypotheses in the Greater Antillean group using covariance ratio effect sizes (CR z-scores).
Supplementary Table 9 Statistical support for alternative modularity hypotheses in the Greater Antillean group using covariance ratio effect sizes (CR z-scores) when 10% of the most extreme species are excluded.

Supplementary Table 10 Net evolutionary rates σ2 of body size and comparisons among biogeographic groups.
Supplementary Table 11 Net evolutionary rates ratio between the front and hind module and comparisons among biogeographic groups.
Supplementary Table 12 Differences in relative evolutionary rates among the major biogeographic groups.
Supplementary Table 13 Variation in relative evolutionary rates of the major biogeographic groups.
Supplementary Table 14 Modularity hypothesis testing in a maximum likelihood framework.
Supplementary Table 15 Statistical support for variable rates models.

Supplementary References

Supplementary information

Supplementary Note 1

Modularity analysis

In addition to the covariance ratio (CR) analyses reported in the main text, we used a maximum likelihood framework in the R package EMMLI[1] to evaluate the support for alternative modularity hypotheses in each of the three major biogeographic groups. In agreement with a recent simulation study that showed a tendency of EMMLI to favor more complex modularity hypotheses[2], we found that the modularity hypothesis that received the highest support in all groups was the configuration with four separate modules (H1; Supplementary Table 5). However, we found that the modularity hypotheses that received the highest CR support in the Primary Mainland, namely H1 with limbs forming one module and girdles two separate modules, also received an AIC support in EMMLI that is statistically indistinguishable from the best model (ΔAIC = 0.70). We thus conclude that the two alternative methods produce broadly consistent results, but diverge in expected aspects in agreement with previous studies[2].

Integration analysis

Given these differences in modularity structure between the major biogeographic groups of *Anolis*, we proceeded to compare the strength of pairwise integration between all four blocks in each of the major biogeographic groups. We used phylogenetic partial least square (PLS) analyses coupled with effect size comparisons (PLS z-scores) following Adams and Collyer[3]. Species coverage between the three major biogeographic groups is uneven (see Fig. 1a) and we detected that this influences the estimated strength of evolutionary integration, but not modularity (denser sampling of a group leads to an increase in the estimated strength of phenotypic integration; Supplementary Fig. 2). We therefore implemented a subsampling approach[4] that equalizes species coverage per group and thus obviates this bias.

Applying this method, we find that the evolutionary integration between fore- and hindlimbs is almost twice as strong as the integration between other modules (Supplementary Fig. 3). Although this seemingly contradicts the prevailing modularity structure across the Anolis, with fore- and hindlimbs being assigned to separate modules (see above), this is explained by the fact that the PLS method maximizes and compares variation along a single dimension, and thus captures higher proportions of the total variation in the limbs compared to the more complex girdles.

In accordance with the distinct modularity structure of the locomotor skeleton in the Primary Mainland clade, we find that the integration between limbs and their respective girdles are generally lower in this clade than in the Greater Antillean group (statistically significant in 49.3% [front-module] and 18.6% [hind-module] of subsampled datasets; Supplementary Fig. 3c and d). For the Secondary Mainland, this was only evident for the front-module (significant in 41.3% of subsampled

datasets; Supplementary Fig. 3c). While the greater morphological disparity in the Greater Antillean group compared to the Secondary Mainland clade was accompanied by a similar overall modularity structure, the four main blocks of the locomotor skeleton exhibited both increased and reduced pairwise integration. In particular, integration between the length of bones of the hindlimb and the shape of the pelvis was stronger in the Greater Antillean group than in the Secondary Mainland clade (significant in 28.4% of subsampled datasets; Supplementary Fig. 3d), but the opposite was true for the integration between the bones of fore- and hindlimbs (significant in 31.9% of subsampled datasets; Supplementary Fig. 3a).

Overall, these results suggest that the differences in morphological disparity between the three groups, including the Greater Antillean and Secondary Mainland groups that share the same pattern of evolutionary modularity, are not accompanied by consistently weaker or stronger covariation structure between the four main blocks of the locomotor skeleton.

Supplementary information

Supplementary Figures

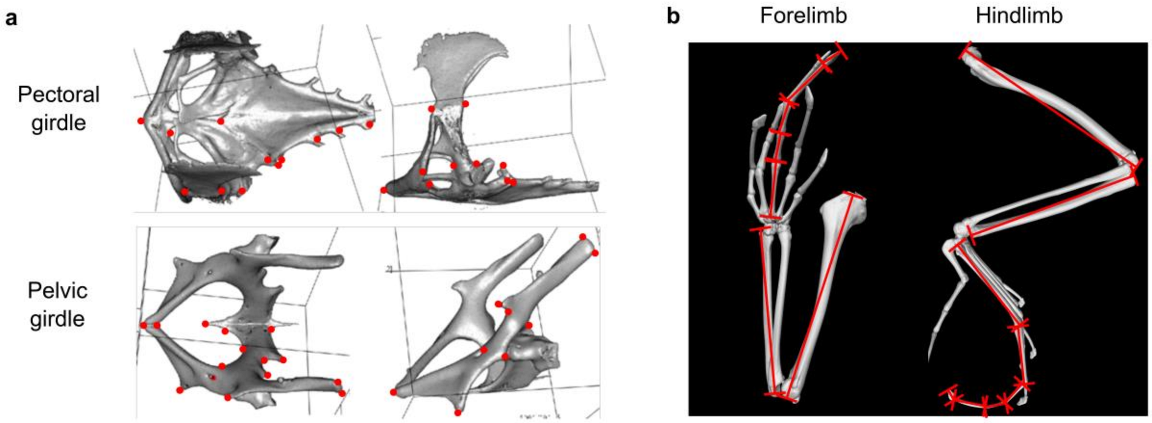

Supplementary Fig. 1 | Quantification of variation in the locomotor skeleton. **a** Placement of 18 3D landmarks on each of the pectoral and pelvic girdles (for detailed description on their exact anatomical position, see Supplementary Table 1). **b** Univariate measurements of the length of 15 individual bones of both fore- and hindlimb.

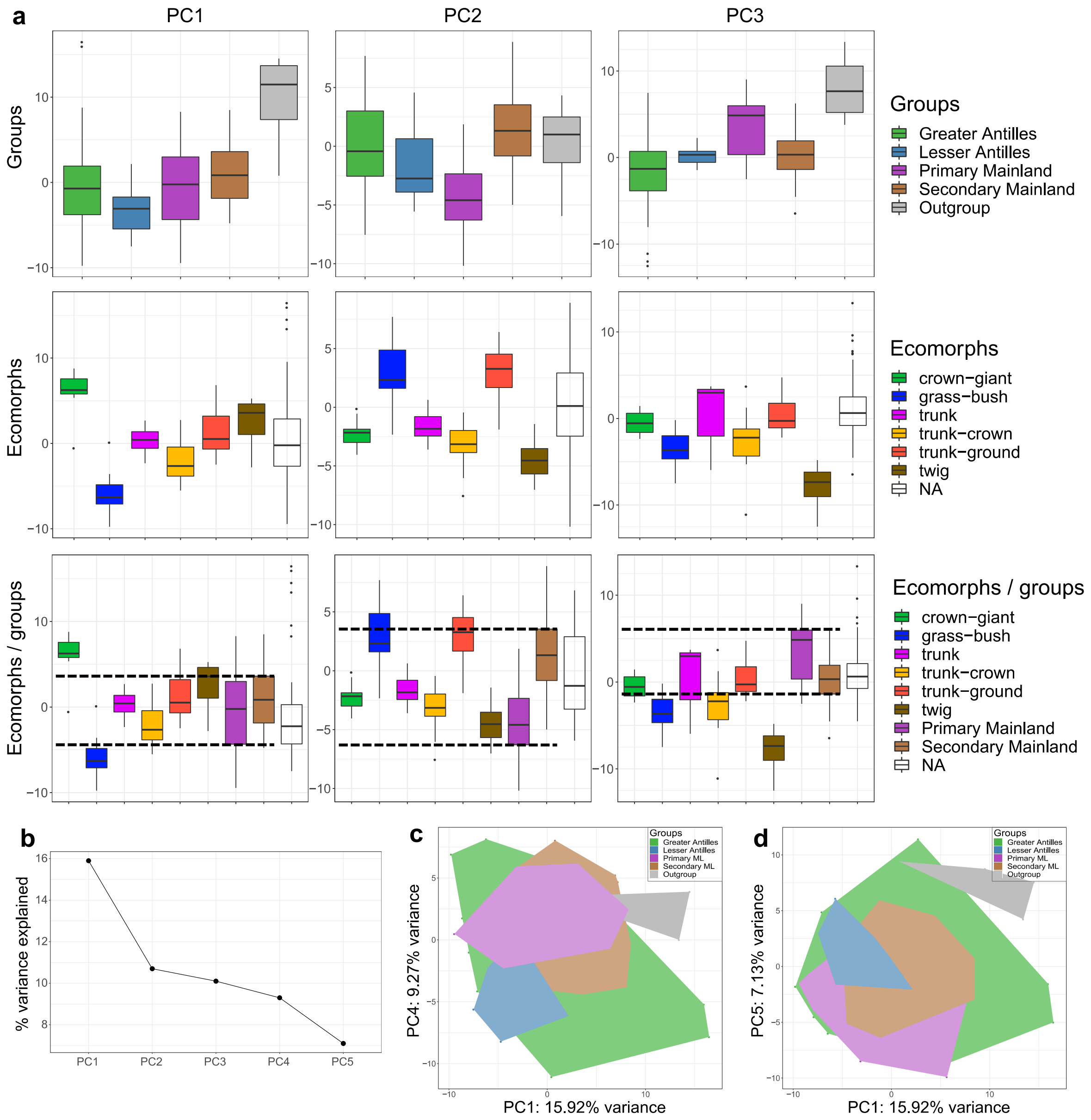

Supplementary Fig. 2 | Distribution of principal component scores by biogeographic group and ecomorph. a Interquartile ranges of the scores of the first, second and third principal components. Boxes are coloured according to biogeographic group (top row), ecomorph (middle row), or both (bottom row). Whiskers indicate the range that lies within 1.5 times of the interquartile ranges and dots show outliers. Note that the two outlier species belonging to the Greater Antillean group that score high on PC1 (top left panel) are *A. porcus* and *A. chamaeleonides*, which are also highlighted in Fig. 1b). The dashed black lines in the bottom row mark the range of PC scores that are occupied by Primary and Secondary Mainland species and visualize which ecomorph classes fall outside this range (crown-giant and grass bush for PC1, and twig for PC3). Sample sizes of each group are n = 110 for Greater Antilles, n = 21 for Lesser Antilles, n = 29 for Primary Mainland, n = 107 for Secondary Mainland, n = 4 for Outgroup, n = 9 for crown-giant, n = 21 for grass-bush,

n = 9 for trunk, n = 21 for trunk-crown, n = 24 for trunk-ground, n = 8 for twig and n = 179 for NA (no ecomorph). **b** Scree plot showing the percentage of variance explained by the five principal components that account for more than 5%. **c,d** The first and fourth, and first and fifth PCs visualizing morphospace occupancy of *Anolis* species colour-coded by biogeographic group.

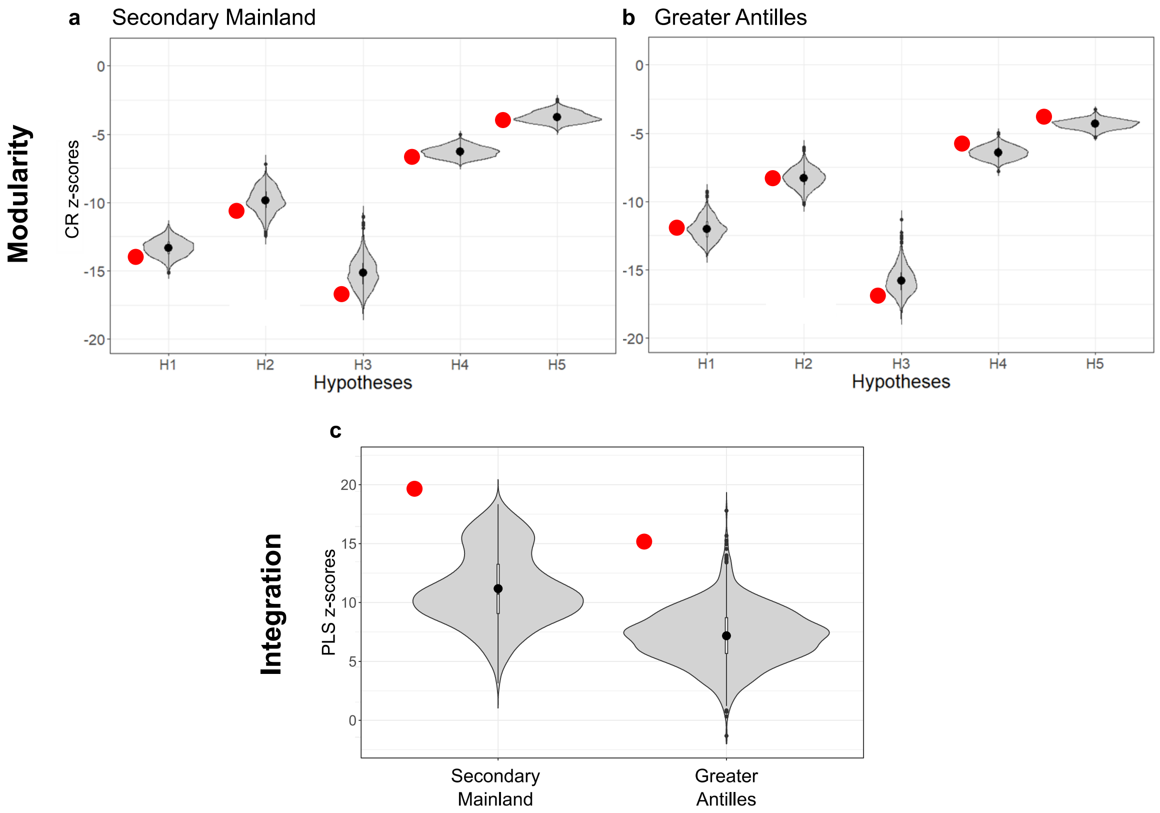

Supplementary Fig. 3 | Negligible effect of subsampling on estimates of modularity, but substantial effect on estimates of integration. a,b Estimation of the strength of modularity (i.e., CR z-scores) in the Secondary Mainland (a) and Greater Antillean (b) groups. **c** Estimation of the strength of integration (i.e., PLS z-scores). Species were subsampled over 1000 iterations to the same proportional coverage as the primary mainland (i.e., 39% of all described species resulting in 55 Greater Antillean and 54 Secondary Mainland species). The strength of modularity for each of the five modularity hypotheses (**a,b**) was estimated in each iteration. Violin plots show the corresponding distribution of n = 1000 iterations in effect sizes in grey (CR and PLS z-scores). Within the violin plots, box plots show the span of the interquartile range with the mean indicated by a horizontal line, whiskers representing 1.5 times the interquartile range and outliers are represented by dots. For comparison, the single effect sizes obtained for the full datasets are shown as red dots.

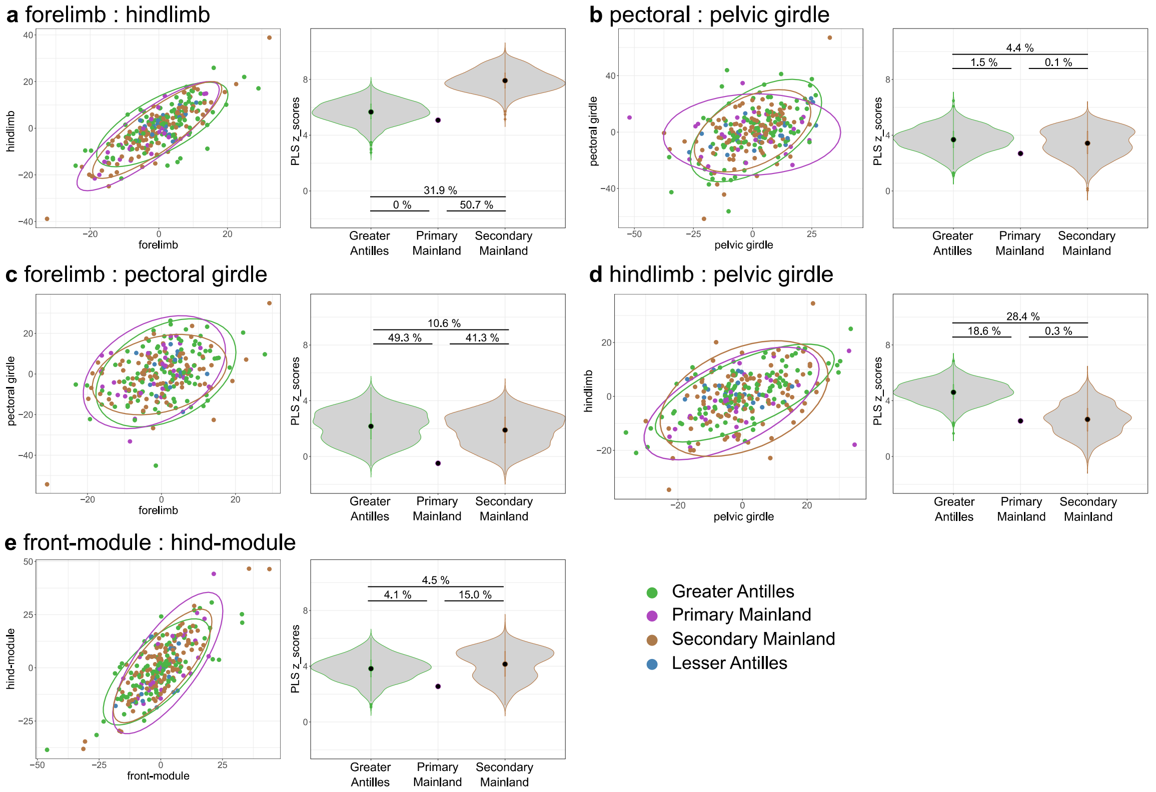

Supplementary Fig. 4 | Patterns of phenotypic integration between the four main blocks of the locomotor skeleton across the major biogeographic groups of *Anolis* lizards. a-e The strength of phenotypic integration was estimated between pairs of the four blocks of the locomotor skeleton and between the front- and the hind-module. For each comparison, left plots show the PLS1 scores for each of the 267 *Anolis* species and ellipses are shown encompassing 95% of species per major biogeographic group. Both the shape and orientation of the ellipses indicate the strength of integration with 'squashed', diagonal ellipses marking strong integration. Right plots of each comparison show the PLS z-score effect sizes derived from subsampling the Greater Antillean and the Secondary Mainland species to attain the same species coverage as the Primary Mainland (i.e., 39%) in 1000 iterations[4]. Violin plots give the distribution of n = 1000 derived PLS z-scores for the subsampled groups, and black dots mark the mean for each distribution. Within the violin plots, box plots show the span of the interquartile range with the mean indicated by a horizontal line and whiskers representing 1.5 times the interquartile range. Percentages provided in these plots give the proportion of subsampled datasets in which the strength of integration was significantly different between two groups (see Methods). Note that standard deviations of PLS z-scores are not plotted, but were accounted for in significance tests.

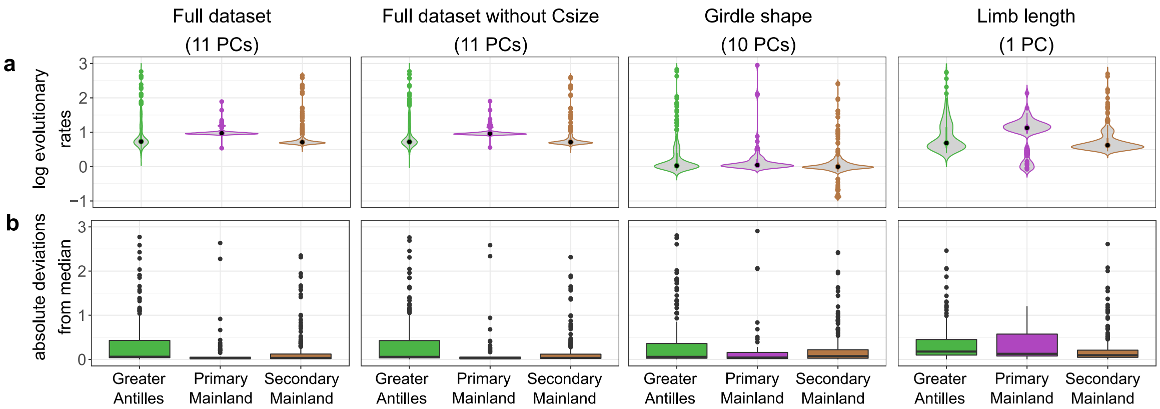

Supplementary Fig. 5 | Variation in Evolutionary rates. a Violin plots showing the spread of log-transformed evolutionary rates per branch for the three major biogeographic groups. **b** Box plots of the absolute deviations from the median. Plots are shown for four different datasets. Across all datasets, the Greater Antillean group has the highest median absolute deviation (MAD) values and the evolutionary rates are significantly more variable in the Greater Antillean compared to the Secondary Mainland group in all datasets except for the girdle shape (see Table S11 and S12 for the statistical results). Within the violin plots in panel **a** and in panel **b**, box plots show the span of the interquartile range with the median indicated by a black dot in panel **a** and a horizontal line in panel **b**, whiskers representing 1.5 times the interquartile range and outliers are represented by dots. The number of branches included in each group are N = 229 for Greater Antilles, N = 58 for Primary Mainland and N = 208 for Secondary Mainland. Abbreviations: Csize, centroid size; PC, principal component.

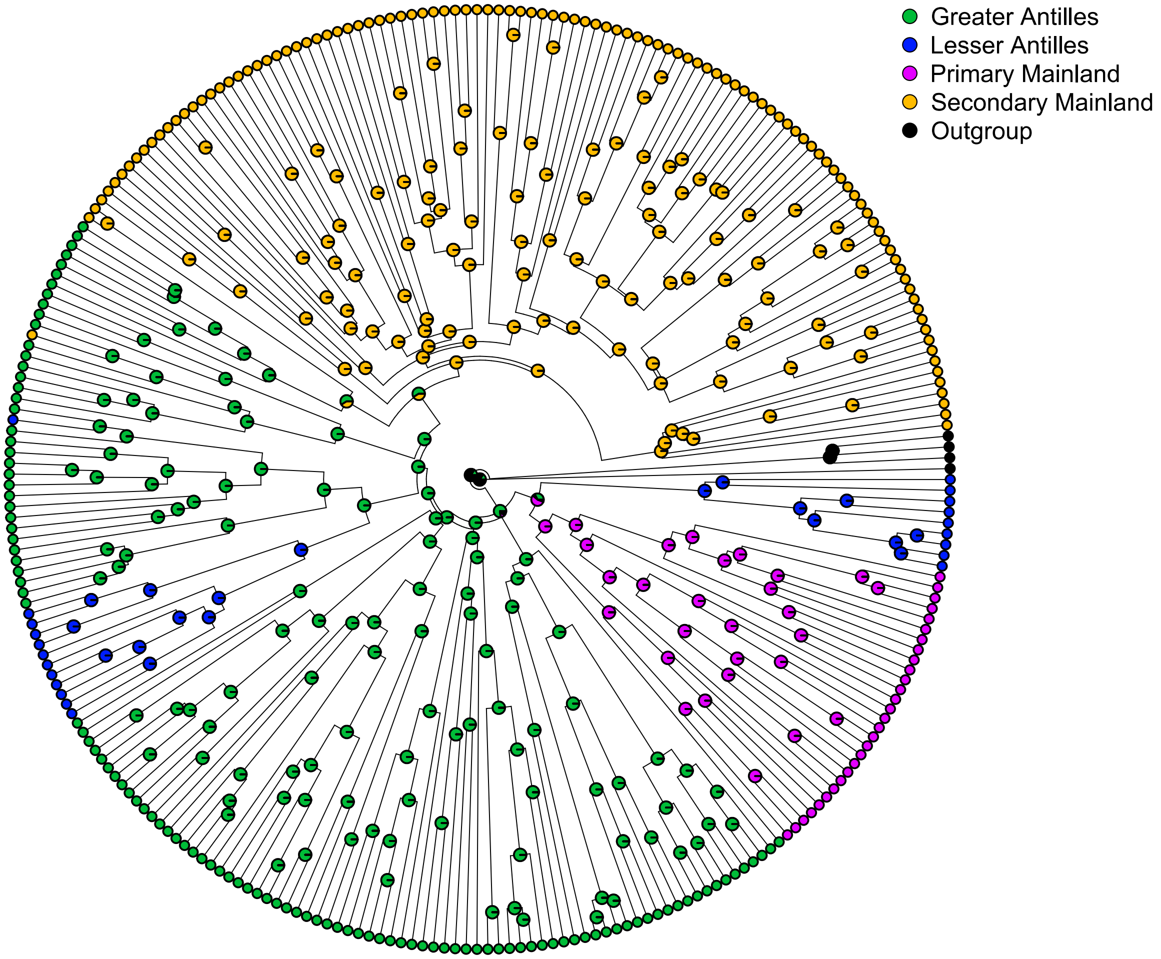

Supplementary Fig. 6 | Ancestral state reconstruction of biogeographic groups. The likely biogeographic state of all ancestral nodes were estimated using the 'ace' function in the R package 'ape'. Biogeographic states were modelled as discrete characters using an 'equal rates' model. A cut-off of ≥ 0.5 scaled likelihood was used to assign ancestral nodes to a given biogeographic state. Pie charts at each node give the scaled likelihood that the ancestor representing this node was a member of a given biogeographic group. Note that only a few nodes at the base of the phylogeny show uncertainties as to which biogeographic group they are assigned. From this ancestral state reconstruction we inferred that the 115 internal nodes are assigned to the Greater Antillean group, 104 nodes to the Secondary Mainland clade, 29 to the Primary Mainland clade and 18 to the Lesser Antillean group. Note that the Lesser Antillean group consists of two clades plus a single species (*A. acutus*) that colonized the Lesser Antilles recently from the Greater Antilles.

Supplementary information

Supplementary Tables

Supplementary Table 1. Anatomical position of landmarks used to quantify the shape of pectoral and pelvic girdles.

Girdle	Landmark	Anatomical position
Pectoral	1	Lateral apex of the primary curvature of the clavicle
	2	Anterior extremity of the clavicle
	3	Anterior-most point of the dorsal edge of the interclavicle
	4	Lateroposterior extremity of the epicoracoid
	5	Lateral extremity of the interclavicle
	6	Dorsoposterior-most point of contact between the clavicle and the scapulocoracoid
	7	Anterior-most point of contact between the suprascapula and the scapula
	8	Posterior-most point of contact between the suprascapula and the scapula
	9	Ventroanterior extremity of the scapular ray
	10	Posterior extremity of the primary coracoid fenestra
	11	Posterior extremity of the scapulacoracoid fenestra
	12	Anteromedial extremity of the presternum
	13	Articulatory point between the intersternum and the first sternal rib
	14	Articulatory point between the intersternum and the second sternal rib
	15	Articulatory point between the intersternum and the third sternal rib
	16	Lateral-most point of the dorsal edge of the coracosternal groove
	17	Dorsal extremity of the dorsolateral process of the presternum
	18	Dorsal extremity of the superior glenoid buttress
Pelvic	1	Anterior extremity of the epipubis
	2	Posterior extremity of the epipubis
	3	Ventrolateral extremity of the pubic tubercle
	4	Ventroposterior extremity of the obturator foramen
	5	Anterior extremity of the proischiadic cartilage
	6	Anterolateral-most point of contact between the proischiadic cartilage and the ischium
	7	Posterior extremity of the thyroid fenestra
	8	Lateral extremity of the thyroid fenestra
	9	Posterolateral-most point of contact between the hypoischium and the ischium
	10	Posterior extremity of the ischiadic tuberosity

11	Anterior-most point of the curvature between the ischiadic tuberosity and the acetabulum
12	Ventroposterior extremity of the acetabulum
13	Anterodorsal extremity of the preacetabular process
14	Dorsal-most point of contact between the ilium and the epiphyseal tuberosity
15	Ventral-most point of contact between the ilium and the epiphyseal tuberosity
16	Anterior extremity of the acetabulum
17	Apex of the anterodorsal curve of the anterior portion of the ilium
18	Anterior-most point of the curvature between the acetabulum and epiphyseal tuberosity

Landmarks for capturing variation in the pectoral girdles were partially adopted from Tinius and Russell[5] and for the pelvic girdle from Tinius *et al.*[6].

Supplementary Table 2. Loadings of the principal components.

PC1		PC2		PC3	
trait	loading	trait	loading	trait	loading
pel_LM1.Y	0.191	pec_LM14.X	0.196	pha1_f_length	0.219
pel_LM2.Y	0.177	pec_LM6.Y	0.186	pha4_f_length	0.200
pel_LM8.X	0.174	pec_LM15.X	0.171	ulna_length	0.193
pel_LM6.Z	0.163	pec_LM7.Y	0.145	pha5_h_length	0.193
pel_LM6.X	0.163	pel_LM17.Z	0.136	pha2_h_length	0.183
pel_LM7.X	0.151	pel_LM10.X	0.133	tibia_length	0.181
pel_LM9.Z	0.148	pel_LM13.Z	0.123	pha2_f_length	0.178
pec_LM7.Y	0.137	pel_LM6.Y	0.122	pha1_h_length	0.177
pec_LM8.Y	0.136	pec_LM4.Z	0.120	pel_LM15.X	0.170
pel_LM14.Z	0.126	pec_LM16.Z	0.118	digit_claw	0.168
pel_LM14.X	-0.130	pec_LM9.Y	-0.129	pec_LM12.Y	-0.105
pel_LM3.Y	-0.132	pec_LM3.Y	-0.135	pec_LM5.Y	-0.118
pel_LM4.Z	-0.145	pec_LM18.Y	-0.135	pel_LM5.Y	-0.123
pel_LM8.Y	-0.147	pec_LM12.X	-0.140	pec_LM16.X	-0.131
pel_LM13.Y	-0.153	pel_LM15.Y	-0.151	pel_LM18.X	-0.132
pel_LM16.Y	-0.154	pec_LM1.X	-0.169	pel_LM7.X	-0.133
pel_LM16.Z	-0.155	pec_LM10.Y	-0.172	pel_LM10.X	-0.137
pel_LM2.X	-0.178	pec_LM6.Z	-0.184	pec_LM5.Z	-0.143
pel_LM17.Y	-0.197	pec_LM11.Y	-0.184	pel_LM11.X	-0.147
pel_LM1.X	-0.199	pel_LM14.Y	-0.206	pel_LM12.X	-0.161

The ten most positive and most negative loadings for each of the first three principal components (PCs) are given. Abbreviations: cort_thick, cortical thickness; LM, landmark; pec, pectoral girdle; pel, pelvic girdle; pha1_f_length, first phalange of the forelimb; pha2_f_length, second phalange of the forelimb; pha4_f_length, forth phalange of the forelimb; pha1_h_length, first phalange of the hindlimb; pha2_h_length, second phalange of the hindlimb; pha5_h_length, fifth phalange of the hindlimb.

Supplementary Table 3. Morphological disparity of girdles (108 girdle shape traits) and comparisons among biogeographic groups.

	Greater Antilles	Primary ML	Secondary ML	Lesser Antilles
Greater Antilles	0.024	0.002	**0.007**	**0.009**
Primary ML	0.460	0.022	**0.005**	**0.007**
Secondary ML	**<0.001**	**0.043**	0.017	0.002
Lesser Antilles	**0.006**	**0.043**	0.420	0.015

The diagonals show Procrustes variances for each biogeographic region (shaded in grey). Off-diagonals show the pairwise absolute differences (top right) and associated *P*-values (bottom left) derived from 999 permutations. Procrustes variances have been calculated based on a phylogenetic model (pgls). Significantly different pairwise comparisons are highlighted in bold. Statistical tests were two-sided and no adjustments for multiple comparisons were applied. Abbreviations: ML, mainland.

Supplementary Table 4. Morphological disparity of limbs (15 limb length traits) and comparisons among biogeographic groups.

	Greater Antilles	Primary ML	Secondary ML	Lesser Antilles
Greater Antilles	0.051	0.004	**0.013**	**0.040**
Primary ML	0.707	0.047	0.009	**0.036**
Secondary ML	**0.036**	0.364	0.038	**0.027**
Lesser Antilles	**0.002**	**0.007**	**0.014**	0.011

The diagonals show Procrustes variances for each biogeographic region (shaded in grey). Off-diagonals show the pairwise absolute differences (top right) and associated *P*-values (bottom left) derived from 999 permutations. Procrustes variances have been calculated based on a phylogenetic model (pgls). Significantly different pairwise comparisons are highlighted in bold. Statistical tests were two-sided and no adjustments for multiple comparisons were applied. Abbreviations: ML, mainland.

Supplementary Table 5. Variation explained by ecomorph.

		Df	R^2-value	F-value	*P*-value	SS
**Full dataset**						
	~ ecomorph	5	0.090	1.708	< 0.001	30592
**Girdle shape**						
	~ ecomorph	5	0.098	1.864	0.003	5.932
**Limbs length**						
	~ ecomorph	5	0.155	3.163	0.002	9.878

We used a phylogenetic generalized least squares regression (PGLS) to quantify the fraction of morphological variation in the locomotor skeleton that is attributable to ecomorph identity. 92 species of the Greater Antilles that are assigned to an ecomorph were subjected to this analysis. The R^2-value represents the proportion of the total variation that is explained by ecomorph. Statistical tests were two-sided and no adjustments for multiple comparisons were applied. Abbreviations: Df, degrees of freedom, SS, sum of squares.

Supplementary Table 6. Statistical support for alternative modularity hypotheses in the Primary Mainland clade using covariance ratio effect sizes (CR z-scores).

	H1	H2	H3	H4	H5
H1	-13.626	-1.612	-1.286	**-4.718**	**-6.511**
H2	0.107	-11.347	0.329	**-3.106**	**-4.899**
H3	0.186	0.744	-11.809	**-3.433**	**-5.225**
H4	**<0.001**	**0.002**	**<0.001**	-6.956	-1.792
H5	**<0.001**	**<0.001**	**<0.001**	0.073	-4.423

The diagonals show CR z-scores for each modularity hypothesis (see Fig. 2b for specific configurations). Off-diagonals show the pairwise, two-sample z-scores (top right) and associated *P*-values (bottom left). Significantly different pairwise comparisons are highlighted in bold. Statistical tests were two-sided and no adjustments for multiple comparisons were applied.

Supplementary Table 7. Statistical support for alternative modularity hypotheses in the Secondary Mainland clade using covariance ratio effect sizes (CR z-scores).

	H1	H2	H3	H4	H5
H1	-13.641	**-2.476**	**2.100**	**-5.218**	**-7.029**
H2	**0.013**	-10.142	**4.576**	**-2.742**	**-4.553**
H3	**0.036**	**<0.001**	-16.609	**-7.318**	**-9.129**
H4	**<0.001**	**0.006**	**<0.001**	-6.265	-1.811
H5	**<0.001**	**<0.001**	**<0.001**	0.070	-3.706

The diagonals show CR z-scores for each modularity hypothesis (see Fig. 2b for specific configurations). Off-diagonals show the pairwise, two-sample z-scores (top right) and associated *P*-values (bottom left). Significantly different pairwise comparisons are highlighted in bold. Statistical tests were two-sided and no adjustments for multiple comparisons were applied.

Supplementary Table 8. Statistical support for alternative modularity hypotheses in the Greater Antillean group using covariance ratio effect sizes (CR z-scores).

	H1	H2	H3	H4	H5
H1	-11.899	**-2.579**	**3.486**	**-4.301**	**-5.729**
H2	**0.010**	-8.253	**6.065**	-1.722	**-3.150**
H3	**<0.001**	**<0.001**	-16.826	**-7.787**	**-9.215**
H4	**<0.001**	0.085	**<0.001**	-5.819	-1.428
H5	**<0.001**	**0.002**	**<0.001**	0.153	-3.800

The diagonals show CR z-scores for each modularity hypothesis (see Fig. 2b for specific configurations). Off-diagonals show the pairwise, two-sample z-scores (top right) and associated *P*-values (bottom left). Significantly different pairwise comparisons are highlighted in bold. Statistical tests were two-sided and no adjustments for multiple comparisons were applied.

Supplementary Table 9. Statistical support for alternative modularity hypotheses in the Greater Antillean group using covariance ratio effect sizes (CR z-scores) when 10% of the most extreme species are excluded.

	H1	H2	H3	H4	H5
H1	-11.742	**-2.576**	**3.648**	**-4.218**	**-5.599**
H2	**0.010**	-8.102	**6.224**	-1.642	**-3.024**
H3	**<0.001**	**<0.001**	-16.899	**-7.866**	**-9.248**
H4	**<0.001**	0.100	**<0.001**	-5.780	-1.381
H5	**<0.001**	**0.002**	**<0.001**	0.167	-3.828

The diagonals show CR z-scores for each modularity hypothesis (see Fig. 2b for specific configurations). Off-diagonals show the pairwise, two-sample z-scores (top right) and associated *P*-values (bottom left). Significantly different pairwise comparisons are highlighted in bold. The dataset used in this analysis contains 99 species from the Greater Antilles. The most extreme species in terms of overall morphology were identified by assessing which each species' distance in PC scores (mean of PC1, PC2 and PC3) from to the mean of all *Anolis* species' PC scores. Statistical tests were two-sided and no adjustments for multiple comparisons were applied.

Supplementary Table 10. Net evolutionary rates *σ*^{2} of body size and comparisons among biogeographic groups.

	Greater Antilles	Primary Mainland	Secondary Mainland	Lesser Antilles
Greater Antilles	715.955	1.186	**2.996**	1.156
Primary ML	0.764	603.856	2.527	1.371
Secondary ML	**<0.001**	0.053	238.972	**3.463**
Lesser Antilles	0.742	0.657	**0.007**	827.601

The diagonals show the net evolutionary rates σ^2 of centroid size which is a proxy for body size. Off-diagonals show the pairwise rate ratios (top right) and associated *P*-values (bottom left) derived from 999 permutations. Significantly different pairwise comparisons are highlighted in bold. Statistical tests were two-sided and no adjustments for multiple comparisons were applied.

Supplementary Table 11. Net evolutionary rates ratio between the front and hind module and comparisons among biogeographic groups.

	Greater Antilles	Primary Mainland	Secondary Mainland	Lesser Antilles
σ^2_{mult} front module	2064.89	1744.58	1882.54	1403.14
σ^2_{mult} hind module	1631.97	1931.44	1537.89	1355.00
Rate ratio (front:hind)	**1.27**	0.90	**1.22**	1.04
P-value	**<0.001**	0.198	**<0.001**	0.796

For each group, the net evolutionary rates σ^2 of the front and hind module, their rate ratio, and the associated *P*-values (derived from 999 permutations) are given. Significantly different pairwise comparisons are highlighted in bold. Statistical tests were two-sided and no adjustments for multiple comparisons were applied.

Supplementary Table 12. Differences in relative evolutionary rates among the major biogeographic groups.

	Kruskal-Wallis test	multiple comparison post hoc tests		
		Greater Antilles vs Primary Mainland	Greater Antilles vs Secondary Mainland	Primary Mainland vs Secondary Mainland
Full dataset (11PCs)	**X^2 = 52.52** **P = <0.001**	**diff = 112.60** **TRUE**	**diff = 40.84** **TRUE**	**diff = 153.44** **TRUE**
Full dataset without Csize (11PCs)	**X^2 = 49.19** **P = <0.001**	**diff = 116.08** **TRUE**	**diff = 32.88** **TRUE**	**diff = 148.96** **TRUE**
Girdle shape (10 PCs)	**X^2 = 34.32** **P = <0.001**	diff = 22.88 FALSE	**diff = 70.35** **TRUE**	**diff = 93.23** **TRUE**
Limb length (1 PC)	**X^2 = 16.63** **P = <0.001**	diff = 49.17 FALSE	**diff = 33.58** **TRUE**	**diff = 82.75** **TRUE**

Relative rates of evolution (log-transformed) have been extracted from the posterior distribution of two independent runs of rjMCMC in BayesTraits and for each branch, and the means of the two runs were inferred. We performed Kruskal-Wallis tests[7] to determine if evolutionary rates differ between the three major groups (df = 2 and two-sided for all tests). For Kruskal-Wallis tests that produced a significant *P*-value, we performed multiple comparison tests using the 'kruskalmc' function from the pgirmess package (version 1.6.9). For these tests, we report the observed difference (diff) and a statement (TRUE or FALSE) on the significance of the observed difference as reported by the 'kruskalmc' test. Significantly comparisons are highlighted in bold. Plots of the distribution of evolutionary rates across branches are shown in Fig. 3a and Supplementary Fig. 4. The number of branches included in each group are N = 229 for Greater Antilles, N = 58 for Primary Mainland and N = 208 for Secondary Mainland.

Supplementary Table 13. Variation in relative evolutionary rates of the major biogeographic groups.

	Levene's test	Tukey post hoc test		
		Greater Antilles vs Primary Mainland	Greater Antilles vs Secondary Mainland	Primary Mainland vs Secondary Mainland
Full dataset (11PCs)	$\boldsymbol{F_{(2,492)}}$ **= 5.85** ***P*** **= 0.003**	diff = -0.16 *P* = 0.056	**diff = -0.14** ***P*** **= 0.006**	diff = 0.02 *P* = 0.960
Full dataset without Csize (11PCs)	$\boldsymbol{F_{(2,492)}}$ **= 6.16** ***P*** **= 0.002**	diff = -0.17 *P* = 0.054	**diff = -0.15** ***P*** **= 0.004**	diff = 0.02 *P* = 0.974
Girdle shape (10 PCs)	$F_{(2,492)}$ = 0.96 *P* = 0.385	NA	NA	NA
Limb length (1 PC)	$\boldsymbol{F_{(2,492)}}$ **= 3.72** ***P*** **= 0.025**	diff = 0.02 *P* = 0.952	**diff = -0.09** ***P*** **= 0.036**	diff = -0.11 *P* = 0.140

Relative rates of evolution (log-transformed) have been extracted from the posterior distribution of two independent runs of rjMCMC in BayesTraits and for each branch, and the means of the two runs were inferred. We performed Levene's tests (two-sided) of equality of variances[8] of the three major groups on the median of the distributions (corresponding to the robust Brown-Forsythe Levene-type procedure[9]). For Levene's tests that produced a significant *P*-value, we performed Tukey post hoc tests (two-sided). Significantly comparisons are highlighted in bold. Plots of the distribution of evolutionary rates across branches are shown in Fig. 3b and Supplementary Fig.4. The number of branches included in each group are N = 229 for Greater Antilles, N = 58 for Primary Mainland and N = 208 for Secondary Mainland.

Supplementary Table 14. Modularity hypothesis testing in a maximum likelihood framework.

Group	Modularity hypothesis	Module structure	AIC	ΔAIC	ML
Primary Mainland	H4. four separate modules	pec : pel : FL : HL	-566.11	0	294.07
	H1. girdles sep vs limbs	pec : pel : (FL,HL)	-565.41	0.70	287.71
	H5. girdles vs limbs sep	(pec,pel) : FL : HL	-565.35	0.76	289.68
Secondary Mainland	H4. four separate modules	pec : pel : FL : HL	15713.58	0	-7845.77
Greater Antilles	H4. four separate modules	pec : pel : FL : HL	47223.98	0	-23600.97

Five conceivable modularity hypotheses were compared using the software EMMLi[1] in each of the major biogeographic groups. For each group, the modularity hypothesis with a ΔAIC value below 2 are reported. The modularity hypothesis with the highest support is shown in bold. Abbreviations: FL, forelimb; HL, hindlimb; ML, maximum likelihood; pec, pectoral girdle; pel, pelvic girdle; sep, separately.

Supplementary Table 15. Statistical support for variable rates models.

Dataset	Run	Marginal log-likelihood variable rates model	Marginal log-likelihood equal rates model	Bayes factor
Full dataset (11PCs)	1	-6481	-7163	1364.53
Full dataset (11PCs)	2	-6476	-7163	1375.54
Full dataset without Csize (11PCs)	1	-6477	-7157	1361.78
Full dataset without Csize (11PCs)	2	-6466	-7155	1378.71
Girdle shape (10 PCs)	1	-5488	-5295	385.54
Girdle shape (10 PCs)	2	-5496	-5295	394.52
Limb length (1 PC)	1	-128	-123	8.68
Limb length (1 PC)	2	-126	-124	4.78

We used Bayes factors calculated from marginal likelihoods and compared models with variable rates to models with rates constrained as being equal across the *Anolis* tree. See Methods for information on how Bayes factors were calculated.

Supplementary information

Supplementary References

1. Goswami, A. & Finarelli, J. A. EMMLi: A maximum likelihood approach to the analysis of modularity. *Evolution* **70**, 1622-1637 (2016).
2. Adams, D. C. & Collyer, M. L. Comparing the strength of modular signal, and evaluating alternative modular hypotheses, using covariance ratio effect sizes with morphometric data. *Evolution* (2019).
3. Adams, D. C. & Collyer, M. L. On the comparison of the strength of morphological integration across morphometric datasets. *Evolution* **70**, 2623-2631 (2016).
4. Dellinger, A. S. *et al.* Modularity increases rate of floral evolution and adaptive success for functionally specialized pollination systems. *Commun Biol* **2** (2019).
5. Tinius, A. & Russell, A. P. Geometric morphometric analysis of the breast-shoulder apparatus of lizards: a test case using Jamaican anoles (Squamata: Dactyloidae). *Anat Rec (Hoboken)* **297**, 410-432 (2014).
6. Tinius, A., Russell, A. P., Jamniczky, H. A. & Anderson, J. S. What is bred in the bone: Ecomorphological associations of pelvic girdle form in greater Antillean Anolis lizards. *J Morphol* **279**, 1016-1030 (2018).
7. Kruskal, W. H. & Wallis, W. A. Use of Ranks in One-Criterion Variance Analysis. *Journal of the American Statistical Association* **47**, 583-621 (1952).
8. Levene, H. in *Contributions to Probability and Statistics* (Stanford University Press, 1960).
9. Brown, M. B. & Forsythe, A. B. Robust Tests for the Equality of Variances. *Journal of the American Statistical Association* **69**, 364-367 (1974).
