# Supplementary material for: Evolution of the locomotor skeleton in Anolis lizards reflects the interplay between ecological opportunity and phylogenetic inertia
Source: Nat Commun. 2021 Mar 9;12:1525. doi: 10.1038/s41467-021-21757-5 (PMC7943571; doi:10.1038/s41467-021-21757-5)
Supplement: Supplementary file 3 — Description of Additional Supplementary Files [file 41467_2021_21757_MOESM3_ESM.pdf]

Description of Additional Supplementary Files

File Name: Supplementary Data 1
Description: Supplementary Data 1 contains the morphometric dataset used in the analyses.

File Name: Supplementary Data 2
Description: Supplementary Data 2 contains the digital object identifiers (DOIs) of the raw scan data for each individual used in this study.
