# Supplementary material for: Evolution of the locomotor skeleton in Anolis lizards reflects the interplay between ecological opportunity and phylogenetic inertia
Source: Nat Commun. 2021 Mar 9;12:1525. doi: 10.1038/s41467-021-21757-5 (PMC7943571; doi:10.1038/s41467-021-21757-5)
Supplement: Supplementary file 6 — Reporting Summary [file 41467_2021_21757_MOESM6_ESM.pdf]

nature research

Corresponding author(s): Nathalie Feiner
Last updated by author(s): Feb 2, 2021

Reporting Summary

Nature Research wishes to improve the reproducibility of the work that we publish. This form provides structure for consistency and transparency in reporting. For further information on Nature Research policies, see our Editorial Policies and the Editorial Policy Checklist.

Statistics

For all statistical analyses, confirm that the following items are present in the figure legend, table legend, main text, or Methods section.

n/a	Confirmed	
☐	☒	The exact sample size (*n*) for each experimental group/condition, given as a discrete number and unit of measurement
☐	☒	A statement on whether measurements were taken from distinct samples or whether the same sample was measured repeatedly
☐	☒	The statistical test(s) used AND whether they are one- or two-sided<br>*Only common tests should be described solely by name; describe more complex techniques in the Methods section.*
☐	☒	A description of all covariates tested
☐	☒	A description of any assumptions or corrections, such as tests of normality and adjustment for multiple comparisons
☐	☒	A full description of the statistical parameters including central tendency (e.g. means) or other basic estimates (e.g. regression coefficient) AND variation (e.g. standard deviation) or associated estimates of uncertainty (e.g. confidence intervals)
☐	☒	For null hypothesis testing, the test statistic (e.g. *F*, *t*, *r*) with confidence intervals, effect sizes, degrees of freedom and *P* value noted<br>*Give P values as exact values whenever suitable.*
☐	☒	For Bayesian analysis, information on the choice of priors and Markov chain Monte Carlo settings
☐	☒	For hierarchical and complex designs, identification of the appropriate level for tests and full reporting of outcomes
☐	☒	Estimates of effect sizes (e.g. Cohen's *d*, Pearson's *r*), indicating how they were calculated

Our web collection on statistics for biologists contains articles on many of the points above.

Software and code

Policy information about availability of computer code

Data collection	micro-CT scans using a GE phoenix v|tome|x m system; data extraction using VGStudio MAX software (version 3.2) and MeshLab (version 2016.12); All details are described in the Method section.
Data analysis	Data analyses were performed in R using the following packages: 'pcaMethods' (version 1.78.0), 'stats' (version 3.6.1), 'geomorph' (version 3.1.3), 'StereoMorph' (version 1.6.3), 'rgl' (version 1.100.30), 'phytools' (version 0.6-99), 'EMMLi' (version 0.0.3), 'vegan' (2.5-6), 'BTprocessR' (version 0.0.1), 'ape' (version 5.3) and 'pgirmess' (version 1.6.9); In addition, we used the softwares 'BayesTraits' (version 3) and 'Tracer' (version 1.5).

For manuscripts utilizing custom algorithms or software that are central to the research but not yet described in published literature, software must be made available to editors and reviewers. We strongly encourage code deposition in a community repository (e.g. GitHub). See the Nature Research guidelines for submitting code & software for further information.

Data

Policy information about availability of data

All manuscripts must include a data availability statement. This statement should provide the following information, where applicable:

- Accession codes, unique identifiers, or web links for publicly available datasets
- A list of figures that have associated raw data
- A description of any restrictions on data availability

Raw scans are available at Morphosource under the project name 'Anolis sp.', project ID P1059. Source data file 1 contains the morphometric dataset used in the analyses. Source data file 2 contains the digital object identifiers (DOIs) of the raw scan data for each individual used in this study.

Field-specific reporting

Please select the one below that is the best fit for your research. If you are not sure, read the appropriate sections before making your selection.

☐ Life sciences ☐ Behavioural & social sciences ☒ Ecological, evolutionary & environmental sciences

For a reference copy of the document with all sections, see nature.com/documents/nr-reporting-summary-flat.pdf

Ecological, evolutionary & environmental sciences study design

All studies must disclose on these points even when the disclosure is negative.

Study description	We used a comparative morphological dataset of Anolis lizards to gain Provides a unique deep-time perspective on a textbook example of adaptive evolution. To address patterns of co-evolution within the locomotor skeleton, we collected geometric morphometric data (landmarks) of girdles and limbs. Using species means, we compared patterns of disparity, evolutionary modularity and integration, and evolutionary rates between major clades of Anolis lizards.
Research sample	Our dataset encompassed 271 species of lizards, 267 of which are Anolis species, and a total of 704 adult males. These are the maximum sample sizes available from museum collections. The morphological dataset comprised 124 traits to fully cover the morphology of the locomotor skeleton (effectively combining landmarks and measurements from all previous studies).
Sampling strategy	Our aim was to cover all species of Anolis lizards. Since the study was limited by collecting scan data, no prior assessment of sample sizes was performed.
Data collection	Data was collected at the Nanoscale Facility of the University of Florida, US, by Nathalie Feiner and Illiam Jackson., using microCT-scanning.
Timing and spatial scale	To cover all the available speciments, data was collected during two visits to the Nanoscale Facility; one in April 2018, and one in July 2018.
Data exclusions	Specimens were excluded from the analyses if their skeletons were not intact. To reduce variation related to sex, we excluded females from our analyses.
Reproducibility	Reproducibility and measurement errors of all individual measurements were determined by the authors, and are reported in the Method section. In brief, reproducibility and measurement errors were quantified through repeat measurements of XX, all of which were blind with respect to speciment ID. Repeatability was tested both within and between subjects (i.e., specimens) and observed (i.e., two researcher). Further, repeatability was ensured by (i) replicating the whole process of data collection, beginning with the import of scanned speciments; (ii) blind repeat measurement of data.
Randomization	The design of our study does not allow for a randomization within experimental units.
Blinding	The design of our study does not allow for a collecting data blindly as the identity of speciments must be known at the time of scanning; however, all landmark and length measurements were collected from scans blindly with respect to the hypotheses tested.

Did the study involve field work? ☐ Yes ☒ No

Reporting for specific materials, systems and methods

We require information from authors about some types of materials, experimental systems and methods used in many studies. Here, indicate whether each material, system or method listed is relevant to your study. If you are not sure if a list item applies to your research, read the appropriate section before selecting a response.

Materials & experimental systems

n/a	Involved in the study	
☒	☐	Antibodies
☒	☐	Eukaryotic cell lines
☒	☐	Palaeontology and archaeology
☐	☒	Animals and other organisms
☒	☐	Human research participants
☒	☐	Clinical data
☒	☐	Dual use research of concern

Methods

n/a	Involved in the study	
☒	☐	ChIP-seq
☒	☐	Flow cytometry
☒	☐	MRI-based neuroimaging

Animals and other organisms

Policy information about studies involving animals; ARRIVE guidelines recommended for reporting animal research

Laboratory animals	The study did not involve laboratory animals, but only specimens from museum collections.
Wild animals	The study did not involve wild animals, but only specimens from museum collections.
Field-collected samples	The study did not involve collection of animals in the field, but only specimens from museum collections.
Ethics oversight	This study does not involve any elements that require specific ethical consideration.

Note that full information on the approval of the study protocol must also be provided in the manuscript.
